# Discordance Between Fasting Plasma Glucose- and HbA1c-Based Glycemic Classifications and Associated Metabolic and Hemodynamic Profiles in Thai Older Adults

**DOI:** 10.3390/life16101598

**Published:** 2026-09-24

**Authors:** Nattaphol Prakobkaew, Orachorn Boonla, Surachat Buddhisa, Surada Satthakarn, Sudarat Boonpitak, Tichanon Promsrisuk, Piyapong Prasertsri

**Affiliations:** 1 Faculty of Allied Health Sciences, Burapha University, Chonburi 20131, Thailand; nattaphol@go.buu.ac.th (N.P.); orachorn@go.buu.ac.th (O.B.); surachat.bu@go.buu.ac.th (S.B.); surada.sa@go.buu.ac.th (S.S.); sudarat.bo@go.buu.ac.th (S.B.); 2 Division of Physiology, School of Medical Sciences, University of Phayao, Phayao 56000, Thailand; tichanon.pr@up.ac.th

**Keywords:** fasting plasma glucose, glycated hemoglobin, glycemic discordance, prediabetes, cardiometabolic risk, older adults, population screening, Thailand

## Abstract

Background: Fasting plasma glucose (FPG) and glycated hemoglobin (HbA1c) reflect different aspects of glycemic regulation and may yield discordant laboratory-defined classifications, particularly among older adults. Methods: This cross-sectional analytical study included 203 community-dwelling Thai adults aged ≥60 years. Single FPG and HbA1c measurements were classified as normal-range, prediabetes-range, or diabetes-range values. Agreement was evaluated using exact agreement, Cohen’s kappa (κ), linear-weighted κ, and symmetry tests. Four binary laboratory-defined phenotypes were compared using age- and sex-adjusted HC3 robust regression. The Benjamini–Hochberg procedure was applied separately across 13 omnibus tests and 39 phenotype-specific contrasts. Results: Exact three-category agreement was 70.9% (unweighted κ = 0.376; 95% CI, 0.247–0.498; weighted κ = 0.486; 95% CI, 0.355–0.601). Binary classifications were discordant in 25.6% of participants; HbA1c-only dysglycemia was numerically more frequent than FPG-only dysglycemia (15.8% vs. 9.9%), but directional asymmetry was not significant (*p* = 0.127). FDR-supported overall phenotype differences involved adiposity, fasting insulin, and lipid measures, whereas hemodynamic measures did not differ. Conclusions: FPG and HbA1c identified overlapping but non-equivalent laboratory-defined groups. Discordant single measurements warrant confirmation and evaluation of biological, analytical, and treatment-related influences; this study does not establish that routine dual testing improves diagnosis or outcomes.

## 1. Introduction

Population aging and the increasing burden of type 2 diabetes have made early identification of dysglycemia an important component of preventive healthcare for older adults. Prediabetes has been associated with increased risks of cardiovascular disease and all-cause mortality in adult populations, although its progression and clinical implications are heterogeneous, particularly among older adults [1]. The identification of dysglycemia also depends partly on the biomarker and diagnostic thresholds employed. Current guidelines recognize fasting plasma glucose (FPG), glycated hemoglobin (HbA1c), and the 2-h plasma glucose concentration during an oral glucose tolerance test (OGTT) as appropriate measures for screening and diagnosing dysglycemia [2]. In community-based settings, FPG and HbA1c are commonly used because they are more practical than an OGTT. Nevertheless, the two markers are not physiologically equivalent and may classify the same individual differently [3].

FPG reflects circulating glucose at a single fasting time point and is determined largely by hepatic glucose production and its suppression by insulin [4]. It is also affected by fasting duration, recent illness, stress, medication use, and short-term biological variation [5]. In contrast, HbA1c reflects cumulative glycemic exposure over the preceding several weeks and incorporates fasting, postprandial, and between-day glucose concentrations [6]. Although HbA1c does not require fasting and generally has lower short-term variability than FPG, its interpretation may be affected by erythrocyte lifespan, anemia, iron status, renal dysfunction, and hemoglobin variants [7]. Consequently, an elevated HbA1c accompanied by normal FPG may reflect postprandial or intermittent hyperglycemia, greater cumulative glycemic exposure, or nonglycemic influences on hemoglobin glycation [8]. Conversely, elevated FPG accompanied by normal HbA1c may reflect isolated fasting hyperglycemia associated with hepatic insulin resistance and impaired early-phase insulin secretion [9].

These differences may be particularly relevant in older adults. HbA1c has been reported to increase with age independently of fasting and post-load glucose, adiposity, and insulin resistance [10], potentially reducing the specificity of fixed HbA1c thresholds in older populations. Red-cell characteristics may modify the association between age and HbA1c, particularly in people with diabetes [11]. Previous research in older adults has shown that substantial proportions of individuals with dysglycemia are identified by FPG or HbA1c alone rather than by both markers [12]. Studies in other populations have also reported limited agreement between FPG- and HbA1c-based classifications, although the magnitude and direction of discordance vary according to age, sex, ethnicity, adiposity, and the distribution of glycemic values within the population [13].

Evidence from Thailand similarly suggests that glycemic tests identify overlapping but non-equivalent groups. Studies comparing HbA1c with the OGTT in high-risk Thai adults found limited agreement [14], particularly for prediabetes, whereas community-based investigations demonstrated that FPG alone may fail to identify a substantial proportion of individuals with post-load dysglycemia [15]. Longitudinal evidence further indicates that combining FPG and HbA1c may improve the identification of Thai individuals at increased risk of progression to diabetes compared with FPG alone [16]. However, most previous Thai studies have focused on high-risk clinical populations, comparing either FPG or HbA1c with the OGTT, or examining the prediction of subsequent diabetes [17]. Less is known about direct agreement between FPG- and HbA1c-based classifications among community-dwelling Thai older adults.

Furthermore, agreement statistics alone may not fully characterize the clinical relevance of discordance. Individuals with HbA1c-only dysglycemia, FPG-only dysglycemia, or concordant dysglycemia may differ in adiposity, insulin metabolism, lipid concentrations, and other cardiovascular risk factors [18]. More broadly, type 2 diabetes is phenotypically heterogeneous, and data-driven subgroups may differ in their risks of complications [19]. Determining whether discordant classifications are accompanied by distinct metabolic characteristics may therefore provide greater insight than treating all individuals above either glycemic threshold as a single homogeneous group.

Accordingly, this study aimed to evaluate agreement between FPG- and HbA1c-based glycemic classifications among community-dwelling Thai older adults using both three-category and binary classification approaches. We additionally defined four phenotypes—concordant normal, HbA1c-only dysglycemia, FPG-only dysglycemia, and concordant dysglycemia—and compared their age- and sex-adjusted metabolic and hemodynamic profiles. We hypothesized that agreement between FPG and HbA1c would be limited and that discordant and concordant dysglycemia phenotypes would exhibit distinct patterns of adiposity, insulin, lipid, and hemodynamic characteristics.

## 2. Materials and Methods

### 2.1. Study Design and Sample Size

This cross-sectional analytical study was conducted at a single center in Mueang District, Chonburi Province, Thailand. A non-probability volunteer sample of 203 older adults was recruited between May 2023 and April 2024. The sample size for the original cross-sectional study was estimated using a single-population proportion formula with finite-population correction. Based on an estimated population of 226,963 adults aged ≥60 years in Chonburi Province, a 95% confidence level (Z = 1.96), a conservative expected proportion of 0.50, and a margin of error of approximately 7.22%, the minimum required sample size was 184 participants. Allowing approximately 10% for incomplete or unusable data yielded a target of 203 participants.

The present study was a secondary analysis of the resulting cross-sectional dataset; no separate a priori sample-size calculation was performed for the agreement and phenotype analyses. The observed laboratory-defined group sizes were not fixed by design; consequently, estimates for the HbA1c-only (*n* = 32) and FPG-only (*n* = 20) groups have lower precision and should be interpreted as exploratory.

### 2.2. Ethical Considerations and Informed Consent

All study procedures were conducted in accordance with the Declaration of Helsinki. The study protocol was approved by the Burapha University Human Ethical Committee (approval no. IRB1-051/2566; 9 May 2023). All participants provided written informed consent before undergoing any study-specific screening or data-collection procedures. Following completion of data collection, the observational study was retrospectively registered with the Thai Clinical Trials Registry to create a publicly accessible study record and improve transparency (TCTR20260106001; registered on 6 January 2026). This retrospective registration did not constitute prospective specification of the analyses reported in the present manuscript.

This study was conducted as part of a broader research project from which a separate analysis of metabolic syndrome and functional exercise capacity was also undertaken [20]. The two studies were based on overlapping participant data and shared selected anthropometric, metabolic, and hemodynamic measurements but addressed distinct research questions and outcomes. The separate study examined the associations of binary metabolic syndrome status and cumulative metabolic syndrome component burden with functional exercise capacity. In contrast, the present study evaluated agreement and discordance between FPG- and HbA1c-based classifications and compared the metabolic and hemodynamic profiles of the resulting glycemic phenotypes among Thai older adults.

### 2.3. Participant Recruitment and Screening

Participants were recruited using a non-probability volunteer sampling approach through advertisements disseminated via community health centers, hospitals, municipal offices, and social media platforms, including Facebook and LINE. Screening and data collection were conducted at the MS 204 Physiotherapy Research Laboratory, Faculty of Allied Health Sciences, Burapha University, Chonburi, Thailand. Because recruitment was conducted through open advertisements, the total number of individuals who viewed or received the advertisements could not be determined.

Eligibility was assessed by the study team using a structured health questionnaire, physical examination, anthropometric measurements, and vital-sign assessment; separate physician assessment or medical documentation was not required for these screening exclusions. Eligible participants were community-dwelling men and women aged ≥60 years who were able to safely complete the required study assessments. Individuals presenting with an infectious or communicable illness at the time of screening, including COVID-19 or influenza, were excluded. Individuals who could not safely complete the required study assessments because of musculoskeletal symptoms, such as knee or low-back pain, or clinically concerning findings identified during screening, including elevated blood pressure (systolic blood pressure [SBP] ≥ 160 mmHg or diastolic blood pressure [DBP] ≥ 100 mmHg) or a recent cardiac arrhythmia, were also excluded.

A total of 219 individuals completed eligibility screening. Of these, 16 were excluded before enrollment because they did not meet the safety requirements for completing the study assessments. Consequently, 203 participants were enrolled and included in the primary analysis. No enrolled participant had missing FPG or HbA1c data; outcome-specific sample sizes are reported where applicable. Physician-diagnosed diabetes mellitus, hypertension, and dyslipidemia, as well as current use of glucose-lowering, antihypertensive, and lipid-lowering medications, were ascertained by self-report. Participants with established or treated diabetes were retained in the primary analysis and classified according to their measured FPG and HbA1c values. Therefore, the resulting categories represent laboratory test-result ranges at the time of assessment rather than confirmed clinical diagnoses or overall glycemic states.

### 2.4. Data Collection Procedures

Following screening, eligible participants were scheduled for laboratory assessment after a 12-h overnight fast, during which only small sips of water were permitted, and were instructed to obtain at least 7 h of sleep. Completion of the required fasting period was verbally confirmed with each participant on the day of blood collection. Participants who reported fasting for less than 12 h were rescheduled for blood collection after completing the required fasting period.

Upon arrival, participants rested quietly for 10 min. BP was then measured, followed by anthropometric and body composition assessments. Venous blood was collected afterward for biochemical analyses (Figure 1).

### 2.5. Blood Pressure Measurement

Following a 10 min rest in a seated position, BP and heart rate (HR) were measured using a digital automatic BP monitor (HEM-7121, Omron Healthcare Co., Ltd., Kyoto, Japan). The cuff was applied to the upper arm with the lower edge positioned approximately 1 inch above the antecubital crease. Two measurements were obtained at 1 min intervals, and the mean of the two readings was used to determine SBP and DBP. Pulse pressure (PP = SBP − DBP) and mean arterial pressure (MAP = DBP + PP/3) were subsequently calculated.

### 2.6. Anthropometric Assessment

Participants were instructed to void their bladder prior to assessment. Anthropometric and body composition parameters were measured using a bioelectrical impedance analyzer (InBody270, InBody Co., Ltd., Daejeon, Republic of Korea). During measurement, participants stood upright with feet positioned shoulder-width apart, arms slightly flexed and abducted, and hands gripping the device handles. Participant sex, age, and height were entered into the system to yield output.

Waist circumference was measured at the midpoint between the lower rib margin and the superior iliac crest at the end of a normal expiration. Hip circumference was measured at the level of maximum gluteal protrusion. The waist-to-hip ratio was subsequently calculated.

### 2.7. Biochemical Analysis

Approximately 10 mL of venous blood was collected into sodium fluoride, ethylenediaminetetraacetic acid (EDTA), and lithium heparin tubes. Fasting plasma glucose (FPG) was measured in sodium fluoride plasma using an enzymatic hexokinase method. Glycated hemoglobin (HbA1c) was measured in EDTA-treated whole blood by high-performance liquid chromatography (HPLC) using a Bio-Rad D-100 Hemoglobin Testing System (Bio-Rad Laboratories, Inc., Hercules, CA, USA). The Bio-Rad D-100 A1c program is listed as an NGSP-certified method, with results standardized to the Diabetes Control and Complications Trial reference system. Insulin was measured by chemiluminescent immunoassay. Total cholesterol and triglycerides were measured using enzymatic colorimetric methods, whereas low-density lipoprotein cholesterol (LDL-C) and high-density lipoprotein cholesterol (HDL-C) were measured using direct assays. All analyses were performed on the day of collection at RIA Laboratory Co., Ltd. (Chonburi, Thailand), an accredited medical laboratory operating in accordance with ISO 15189 and ISO 15190 standards [21,22].

The laboratory maintained internal quality control (IQC) and participated in an external quality assessment (EQA) program for HbA1c. In the available March 2025 IQC documentation, two control levels with laboratory means of 5.32% and 9.78% yielded coefficients of variation of 1.46% and 1.72%, respectively, based on 31 measurements at each level. The available 2025 EQA report identified the analyte as HbA1c (NGSP) and the analytical platform as the Bio-Rad D-100 HPLC system. For the reported EQA specimen, the laboratory obtained a value of 6.85%, compared with a peer-group mean of 7.04% (Z-score = −1.67), with no warning designation. These records document the laboratory’s subsequent performance on the analytical platform but were not contemporaneous with the measurements of the study samples.

Participant-specific chromatograms and systematic records of abnormal hemoglobin-fraction flags were not retained in the research dataset and were therefore unavailable for retrospective review. Hemoglobin typing was not performed as part of the study, and no participant was excluded on the basis of a known or recorded hemoglobin variant. Hematological indices, hemoglobin concentration, iron status, renal function, transfusion history, erythrocyte-turnover indicators, and hemoglobin-variant status were not comprehensively available in the analytical dataset.

### 2.8. Statistical Analysis

The parent research protocol specified the collection of FPG, HbA1c, and the metabolic and hemodynamic measurements used in the present study. However, the agreement analyses, laboratory-defined concordance phenotypes, phenotype-based regression models, multiplicity correction, and sensitivity analyses reported herein were developed for this secondary analysis and were not prospectively registered. Accordingly, these analyses should be considered exploratory.

Distributional characteristics were examined in the pooled sample and within each of the four laboratory-defined phenotypes using histograms, Q-Q plots, and the Shapiro-Wilk test. Continuous variables are presented as medians with interquartile ranges (IQRs), and categorical variables as numbers and percentages. Regression diagnostics included residual distributions, residual-versus-fitted plots, leverage, and Cook’s distance. Because insulin and triglycerides were right-skewed, natural-log-transformed sensitivity models were also fitted.

Glycemic status was classified separately using FPG and HbA1c. For the three-category analysis, FPG values were categorized as normal (<100 mg/dL), prediabetes-range (100–125 mg/dL), or diabetes-range (≥126 mg/dL), whereas HbA1c values were categorized as normal (<5.7%), prediabetes-range (5.7–6.4%), or diabetes-range (≥6.5%).

Agreement between the FPG- and HbA1c-based three-category classifications was assessed using exact percentage agreement, unweighted Cohen’s kappa (κ), and linear-weighted Cohen’s κ. Confidence intervals for all κ coefficients were estimated using 10,000 participant-level nonparametric bootstrap resamples with percentile 95% intervals and random seed 20260912. Bowker’s asymptotic test assessed symmetry of three-category disagreement. Because some discordant cells were sparse, symmetry was additionally evaluated by a conditional permutation test that randomly reversed discordant-pair directions within each category pair using 100,000 permutations, with a random seed of 20260912.

For the binary analysis, prediabetes-range and diabetes-range values were combined into a single dysglycemia category. Binary agreement was evaluated using exact agreement and Cohen’s κ, while the continuity-corrected McNemar test was used to assess directional asymmetry between FPG- and HbA1c-based classifications. Four glycemic phenotypes were subsequently defined: concordant normal, HbA1c-only dysglycemia, FPG-only dysglycemia, and concordant dysglycemia.

Differences in metabolic and hemodynamic profiles across the four phenotypes were examined using multivariable linear regression with HC3 heteroscedasticity-consistent standard errors. The concordant-normal phenotype was the reference category. Age and sex were selected a priori as common demographic covariates that could affect the glycemic markers and outcomes without being consequences of the laboratory-defined phenotypes. The primary models were deliberately parsimonious because the two discordant groups were small and numerous correlated covariates could overfit the models and reduce precision. Adiposity measures were primary outcomes and were therefore not simultaneously treated as routine covariates. Medication use was examined in outcome-specific sensitivity models. Regression coefficients (B), 95% CIs, and *p*-values are reported, and omnibus Wald tests evaluated the phenotype indicators. FPG, HbA1c, and glucose-derived insulin-resistance indices were excluded from the outcome family to avoid mathematical coupling.

The Benjamini–Hochberg procedure was applied separately across the 13 omnibus phenotype tests and across all 39 phenotype-versus-reference contrasts. FDR-adjusted *q*-values < 0.05 were considered statistically significant. These two multiplicity families were interpreted separately, and phenotype-specific contrasts were considered exploratory.

Sensitivity analyses (i) excluded all participants with self-reported diabetes and/or glucose-lowering medication; (ii) added glucose-lowering medication to adiposity and insulin models, antihypertensive medication to hemodynamic models, and lipid-lowering medication to lipid models; and (iii) excluded participants with diabetes-range FPG or HbA1c values. The latter analysis compared normal-range and prediabetes-range laboratory values. Natural-log-transformed insulin and triglyceride models were also examined. *p*-values < 0.05 were considered nominally significant before multiplicity correction. Data management and descriptive analyses used IBM SPSS Statistics for Windows, version 25.0 (IBM Corp., Armonk, NY, USA). Agreement analyses, HC3 models, bootstrap intervals, permutation testing, and FDR correction used Python 3.10 (Python Software Foundation, Wilmington, DE, USA), with SciPy version 1.8.0 (SciPy community) and statsmodels version 0.13.1 (statsmodels developers).

## 3. Results

### 3.1. Participant Characteristics

A total of 219 individuals completed eligibility screening. Of these, 16 were excluded before enrollment because they did not meet the safety requirements for completing the study assessments: elevated BP identified during screening (*n* = 10), knee pain (*n* = 1), low-back pain (*n* = 1), and recent cardiac arrhythmia (*n* = 4). Consequently, 203 participants were enrolled and included in the primary analysis (Figure 2). No participant was excluded for missing FPG or HbA1c data; outcome-specific sample sizes are reported where applicable.

The analytical sample included 167 women (82.3%) and 36 men (17.7%), with a median age of 66.0 years (IQR, 63–70 years). Self-reported physician-diagnosed diabetes was present in 22 participants (10.8%), and 14 (6.9%) used glucose-lowering medication; all 14 medication users were among those with known diabetes. Dyslipidemia was reported by 76 participants (37.4%), with 52 (25.6%) using lipid-lowering medication. Hypertension was reported by 63 participants (31.0%), and 44 (21.7%) used antihypertensive medication. The sample was not selected by probability sampling, and its laboratory-defined proportions should not be interpreted as population prevalence estimates or as representative of all older adults in Chonburi Province or Thailand.

Based on the binary FPG–HbA1c classification, 114 participants (56.2%) were classified as concordant normal, 32 (15.8%) as having HbA1c-only dysglycemia, 20 (9.9%) as having FPG-only dysglycemia, and 37 (18.2%) as having concordant dysglycemia.

Descriptive metabolic and hemodynamic characteristics are shown in Table 1. The observed group sizes arose from the laboratory classifications rather than design quotas. Known diabetes occurred in 0/114, 2/32, 2/20, and 18/37 participants, and glucose-lowering medication use in 0/114, 2/32, 0/20, and 12/37 across the concordant-normal, HbA1c-only, FPG-only, and concordant-dysglycemia groups, respectively. Antihypertensive medication use was 14/114, 12/32, 4/20, and 14/37, and lipid-lowering medication use was 18/114, 14/32, 6/20, and 14/37, respectively.

### 3.2. Agreement Between FPG- and HbA1c-Based Three-Category Classifications

Cross-classification of the single-measurement FPG- and HbA1c-based laboratory ranges is presented in Table 2 and illustrated in the alluvial diagram (Figure 3) and threshold scatterplot (Figure 4). Of 203 participants, 144 had identical three-category classifications, corresponding to exact agreement of 70.9%.

Unweighted Cohen’s κ was 0.376 (95% percentile-bootstrap CI, 0.247–0.498), and linear-weighted κ was 0.486 (95% CI, 0.355–0.601). Thus, observed agreement was higher than agreement beyond chance.

Among 59 participants with discordant three-category classifications, HbA1c assigned a higher range than FPG in 37, and FPG assigned a higher range in 22. The asymptotic Bowker test was not significant (chi-square = 5.599, *p* = 0.133). The conditional permutation test likewise did not show significant directional asymmetry (*p* = 0.089), although the difference from the asymptotic result illustrates the effect of sparse discordant cells.

### 3.3. Agreement Between FPG- and HbA1c-Based Binary Classifications

When prediabetes-range and diabetes-range values were combined into a single laboratory-defined dysglycemia category, exact binary agreement was 74.4%, and Cohen’s κ was 0.404 (95% percentile-bootstrap CI, 0.268–0.533).

Binary classifications were discordant in 52 participants (25.6%; Table 3). HbA1c-only dysglycemia was observed in 32 participants (15.8%), whereas FPG-only dysglycemia was observed in 20 participants (9.9%). Although HbA1c-only dysglycemia was more frequent than FPG-only dysglycemia, the continuity-corrected McNemar test did not demonstrate statistically significant directional asymmetry (χ^2^ = 2.327, *p* = 0.127).

### 3.4. Age- and Sex-Adjusted Metabolic and Hemodynamic Profiles Across Laboratory-Defined Glycemic Phenotypes

Age- and sex-adjusted metabolic and hemodynamic profiles are presented in Table 4. After FDR correction across 13 omnibus tests, overall phenotype differences were supported for BMI, waist circumference, waist-to-hip ratio, fasting insulin, total cholesterol, triglycerides, LDL-C, and HDL-C; no hemodynamic outcome differed (all *q* ≥ 0.330). These are adjusted associations and should not be interpreted as effects caused by phenotype membership.

After separate FDR correction across 39 phenotype-specific contrasts, the FPG-only phenotype had higher BMI (*q* = 0.044), waist circumference (*q* = 0.003), and waist-to-hip ratio (*q* = 0.039) than the concordant-normal phenotype. Its total-cholesterol contrast did not remain significant (*q* = 0.062).

The concordant-dysglycemia phenotype had higher BMI (*q* < 0.001), waist circumference (*q* < 0.001), waist-to-hip ratio (*q* < 0.001), fasting insulin (*q* = 0.003), and triglycerides (*q* = 0.036), and lower total cholesterol (*q* = 0.005) and HDL-C (*q* = 0.003), than the concordant-normal phenotype. No LDL-C contrast remained significant after contrast-level FDR correction.

The HbA1c-only phenotype had higher triglycerides than the concordant-normal phenotype after contrast-level correction (*q* = 0.036). Other HbA1c-only contrasts were not FDR significant. Because the discordant groups were small and unbalanced, these phenotype-specific estimates have limited precision and remain exploratory.

### 3.5. Sensitivity Analyses

Excluding 20 participants with diabetes-range FPG or HbA1c values left 183 participants: concordant normal *n* = 114, HbA1c-only prediabetes-range *n* = 32, FPG-only prediabetes-range *n* = 19, and concordant prediabetes-range *n* = 18. Exact agreement was 72.1%, and Cohen’s κ was 0.236 (95% percentile-bootstrap CI, 0.078–0.388). The McNemar test was not significant (*p* = 0.093). FDR-supported overall phenotype differences remained for BMI (*q* = 0.009), waist circumference (*q* < 0.001), waist-to-hip ratio (*q* = 0.034), insulin (*q* = 0.020), total cholesterol (*q* = 0.013), and triglycerides (*q* = 0.024).

After contrast-level FDR correction in this restricted sample, the concordant prediabetes-range group retained higher BMI (B = 2.86 kg/m^2^; 95% CI, 1.18–4.54; *q* = 0.014) and waist circumference (B = 8.83 cm; 95% CI, 4.14–13.52; *q* = 0.010), and the FPG-only group retained higher waist circumference (B = 7.72 cm; 95% CI, 3.09–12.35; *q* = 0.014). Thus, the overall pattern was not driven solely by diabetes-range values, although several contrasts attenuated.

Excluding all 22 participants with known diabetes and/or glucose-lowering medication left 181 participants (phenotype sizes 114, 30, 18, and 19). Three-category exact agreement was 72.9%; unweighted κ was 0.279 (95% CI, 0.119–0.431) and linear-weighted κ was 0.344 (95% CI, 0.165–0.503). Binary exact agreement was 73.5%, with κ 0.272 (95% CI, 0.115–0.426). FDR-supported overall phenotype differences remained for BMI, waist circumference, waist-to-hip ratio, insulin, total cholesterol, and triglycerides, but not LDL-C or HDL-C.

In outcome-specific medication-adjusted models, FDR-supported overall differences remained for BMI, waist circumference, waist-to-hip ratio, insulin, total cholesterol, triglycerides, LDL-C, and HDL-C, whereas no hemodynamic outcome differed. Medication adjustment did not remove the overall metabolic associations, but residual confounding by drug class, dose, duration, adherence, healthcare use, disease history, and unmeasured factors remains possible. The available medication field did not consistently record drug class, dose, treatment duration, adherence, or treatment changes; therefore, specific antidiabetic therapies could not be compared reliably.

Insulin showed marked right skewness (outcome skewness 3.44; residual skewness 2.99), and triglycerides showed moderate right skewness (1.08 and 0.88, respectively). No model had Cook’s distance > 1; 8 insulin and 10 triglyceride observations exceeded the screening threshold of 4/*n* (maximum Cook’s distances 0.288 and 0.098). Overall phenotype effects persisted for log insulin (*p* < 0.001) and log triglycerides (*p* = 0.001), supporting the main overall findings while indicating sensitivity to influential observations.

## 4. Discussion

FPG and HbA1c identified overlapping but non-equivalent laboratory-defined groups among community-dwelling Thai older adults. Exact agreement was 70.9% for the three-category, and 74.4% for the binary classification, yet agreement beyond chance was modest. Approximately one-quarter of binary classifications were discordant. FDR-supported adjusted associations were observed mainly for adiposity, fasting insulin, and lipid measures, whereas hemodynamic measures did not differ. These associations persisted in several sensitivity analyses but should not be interpreted as phenotype effects because medication exposure, adiposity, healthcare use, disease history, and unmeasured factors may contribute.

The observed discordance is consistent with FPG and HbA1c reflecting related but distinct dimensions and time frames of glycemic regulation [5]. However, HbA1c-only elevation should not be assumed to represent occult or postprandial hyperglycemia. It may also reflect erythrocyte lifespan, anemia, iron deficiency, renal dysfunction, recent transfusion, hemoglobin variants, or assay-specific effects. Conversely, isolated fasting hyperglycemia may reflect impaired suppression of hepatic glucose production, hepatic insulin resistance, and early-phase β-cell dysfunction, without a corresponding elevation in longer-term average glycemic exposure [23]. The present data cannot quantify the relative glycemic and nonglycemic contributions.

Age may further modify the relationship between the two markers. Previous studies have reported that HbA1c tends to increase with age even after accounting for fasting and post-load glucose, adiposity, and insulin resistance [10]. Age-related differences in red-cell characteristics may also alter HbA1c independently of glycemia [11]. In a study of older adults, substantial proportions of individuals with prediabetes or diabetes were identified by FPG or HbA1c alone rather than by both markers, with the extent of disagreement varying by sex and race or ethnicity [12]. Therefore, threshold-based classification using either marker alone may be particularly sensitive to the physiological heterogeneity of older populations.

The magnitude of agreement was lower than in a previous Thai tertiary-hospital study of 4094 individuals, which reported 89.5% agreement and a κ of 0.58 [24], and lower than the weighted κ of 0.55 reported in a population-based Vietnamese study of 3523 adults [3]. A population-based study of older adults in New Mexico likewise found that HbA1c and FPG identified overlapping but non-equivalent groups, with limited agreement between the markers [25]; incomplete concordance has also been reported among Black South African adults [26]. Differences in age, community versus clinical recruitment, glycemic spectrum, established diabetes, medication exposure, assay characteristics, and classification definitions may explain between-study variation; these differences should not be attributed to ethnicity alone. The contribution of the present study is its focus on community-dwelling Thai older adults and the comparison of metabolic and hemodynamic characteristics across four concordance phenotypes, rather than the first demonstration of FPG-HbA1c discordance.

HbA1c-only dysglycemia was numerically more common than FPG-only dysglycemia, although the directional difference was not statistically significant. The HbA1c-only phenotype retained higher triglyceride concentrations after contrast-level FDR correction. This association does not establish that the phenotype caused the metabolic difference, and the HbA1c elevation may partly reflect nonglycemic influences. Iron deficiency can increase HbA1c independently of glycemia [27]. Hemoglobin E may also affect HbA1c interpretation depending on the analytical method [28,29]. According to the NGSP assay-interference table, the Bio-Rad D-100 A1c program shows no clinically significant interference from common heterozygous HbC, HbS, HbE, or HbD traits [29]. Participant-level hemoglobin typing was not performed. Hematological indices, hemoglobin concentration, iron status, renal function, transfusion history, and erythrocyte-turnover indicators were also not comprehensively available. Therefore, variant-related, hematological, renal, and other nonglycemic influences on individual HbA1c values cannot be excluded, and HbA1c-only dysglycemia should not be interpreted as direct evidence of postprandial or otherwise unrecognized hyperglycemia.

The FPG-only phenotype retained higher BMI, waist circumference, and waist-to-hip ratio after contrast-level FDR correction. This pattern is compatible with an adiposity-related metabolic phenotype involving hepatic insulin resistance and fasting hepatic glucose production [30,31]. Previous longitudinal evidence also suggests that FPG-only and HbA1c-only prediabetes may not carry identical risks for subsequent outcomes [32]. Nevertheless, the cross-sectional data cannot distinguish confounding, mediation, shared biological variation, an early stage of dysglycemia, or short-term variation in fasting glucose. Total cholesterol did not remain significant for this contrast after multiplicity correction.

The concordant-dysglycemia group retained higher BMI, waist circumference, waist-to-hip ratio, fasting insulin, and triglycerides and lower total cholesterol and HDL-C after contrast-level correction. These laboratory-defined findings may be compatible with more extensive disturbances across fasting and cumulative glycemic regulation [33], but they do not establish prognosis or justify formal risk stratification. Established diabetes and medication use were concentrated in this group. Longitudinal Thai studies have associated higher FPG and HbA1c values with subsequent diabetes risk [16,34]; however, prospective outcome data specific to the present cohort are needed.

The lipid findings require caution. Medication-adjusted models retained overall phenotype differences, but no LDL-C contrast survived correction across the 39 contrasts. Lower total cholesterol in concordant dysglycemia should not be interpreted as favorable or as caused by phenotype membership. Lipid-lowering therapy, medication class and adherence, dietary fat composition, healthcare utilization, underlying illness, and other unmeasured factors may have influenced the observed lipid profiles [35,36]; the available treatment detail was insufficient for reliable drug-specific analyses.

The absence of significant phenotype differences in BP and HR after FDR correction warrants careful interpretation. In the present study, between-phenotype differences were more evident for adiposity, insulin, and lipid measures than for resting BP or HR. Because the broader research project also included functional exercise-capacity assessments [20], individuals with screening SBP ≥ 160 mmHg or DBP ≥ 100 mmHg were excluded as a safety precaution. Although this criterion was unrelated to glycemic phenotype or the hemodynamic outcomes examined in the present analysis, it may have restricted the upper range of BP and reduced the ability to detect between-phenotype differences. Antihypertensive treatment, measurement variability, and the relatively small discordant groups may have further attenuated these differences. Therefore, the null hemodynamic findings should not be interpreted as evidence that glycemic discordance is unrelated to long-term cardiovascular risk and may not be generalizable to older adults with severe or uncontrolled hypertension.

Exact agreement and κ answer different questions. Exact agreement counts identical classifications and was strongly influenced by the large concordant-normal group (114 of 203). κ discounts agreement expected from the uneven marginal distributions, so a substantial part of the observed 71–74% agreement was expected by chance. The κ of 0.236 after excluding diabetes-range values further indicates limited agreement around the normal-versus-prediabetes threshold. These findings do not demonstrate that routine dual testing is superior for all older adults because no OGTT reference standard, repeated measurements, prospective outcomes, patient benefit, resource use, or cost-effectiveness was assessed. Instead, discordant single screening results may warrant repeat measurement and evaluation of factors affecting either marker, with additional testing guided by clinical context and existing recommendations [2].

Strengths include within-person measurement of FPG and HbA1c, three-category and binary agreement analyses, weighted and unweighted κ with bootstrap intervals, asymptotic and permutation symmetry tests, HC3 robust regression, separate FDR control for omnibus tests and contrasts, outcome-specific medication adjustment, log-transformed sensitivity models, and analyses excluding established diabetes or diabetes-range values.

Several limitations should be acknowledged. The cross-sectional design precludes causal or prognostic conclusions. FPG and HbA1c were measured once, so the categories are screening-based laboratory ranges rather than confirmed diagnoses, and neither marker was an independent reference standard. An OGTT was not performed. Hematological indices, hemoglobin concentration, iron status, renal function, transfusion history, erythrocyte-turnover indicators, and hemoglobin-variant status were not comprehensively available. Participant-specific chromatograms and systematic records of abnormal hemoglobin-fraction flags were unavailable for retrospective review. These factors can influence HbA1c interpretation, particularly in populations in which anemia or hemoglobin variants are prevalent [28,37]. Moreover, the available IQC and EQA documentation was obtained in 2025 and therefore did not constitute contemporaneous quality-control records for the study samples. Detailed information regarding medication class, dose, duration, and adherence was also unavailable. The non-probability, single-center volunteer recruitment strategy may have introduced selection bias, as individuals who responded to community- and social media-based advertisements may differ from the broader population of older adults. The recruitment pathways and voluntary nature of participation may have contributed to the predominance of women, who comprised 82.3% of the sample. Consequently, the findings may not be generalizable to men, institutionalized older adults, older adults in other regions of Thailand, or populations with different levels of healthcare access. Unequal phenotype sizes reduced precision and increased sensitivity to influential observations. Although multiplicity was controlled, the phenotype-specific contrasts remain exploratory. Residual confounding by adiposity, treatment, disease history, and other factors cannot be excluded.

Overall, discordance persisted after exclusion of diabetes-range values and after exclusion of known or treated diabetes. The observed metabolic associations were partly robust to medication adjustment and outcome transformation, but they do not establish causal phenotypes. Longitudinal studies with repeated glycemic measurements, OGTT, hematological and renal assessment, verified medication exposure, and clinical outcomes are required.

## 5. Conclusions

Single FPG and HbA1c measurements provided overlapping but non-equivalent laboratory-defined classifications among community-dwelling Thai older adults. Approximately one-quarter of binary classifications were discordant, and agreement beyond chance remained limited in sensitivity analyses. Adjusted metabolic differences were observed across phenotypes, but the cross-sectional design, treatment exposure, adiposity, analytical influences, and residual confounding preclude causal or diagnostic interpretation.

FPG and HbA1c should therefore be treated as non-interchangeable laboratory indicators. Discordant results may warrant repeat measurement and evaluation of biological, hematological, analytical, and treatment-related factors rather than immediate assignment of a definitive diagnosis. The present study cannot determine whether routine dual testing improves diagnostic accuracy, risk prediction, patient outcomes, or cost-effectiveness. Additional testing should be guided by clinical context and established recommendations.

## Figures and Tables

**Figure 1:**
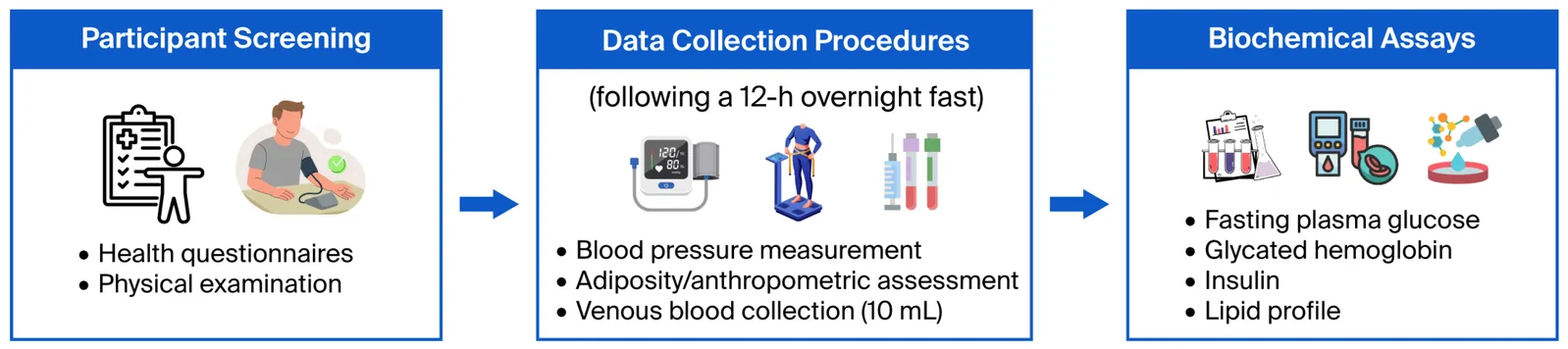
Study protocol, including participant screening, data-collection procedures, and biochemical analysis. The figure was created by the authors using Canva. The icons were sourced from Canva’s content library and used in accordance with the Canva Content License Agreement.

**Figure 2:**
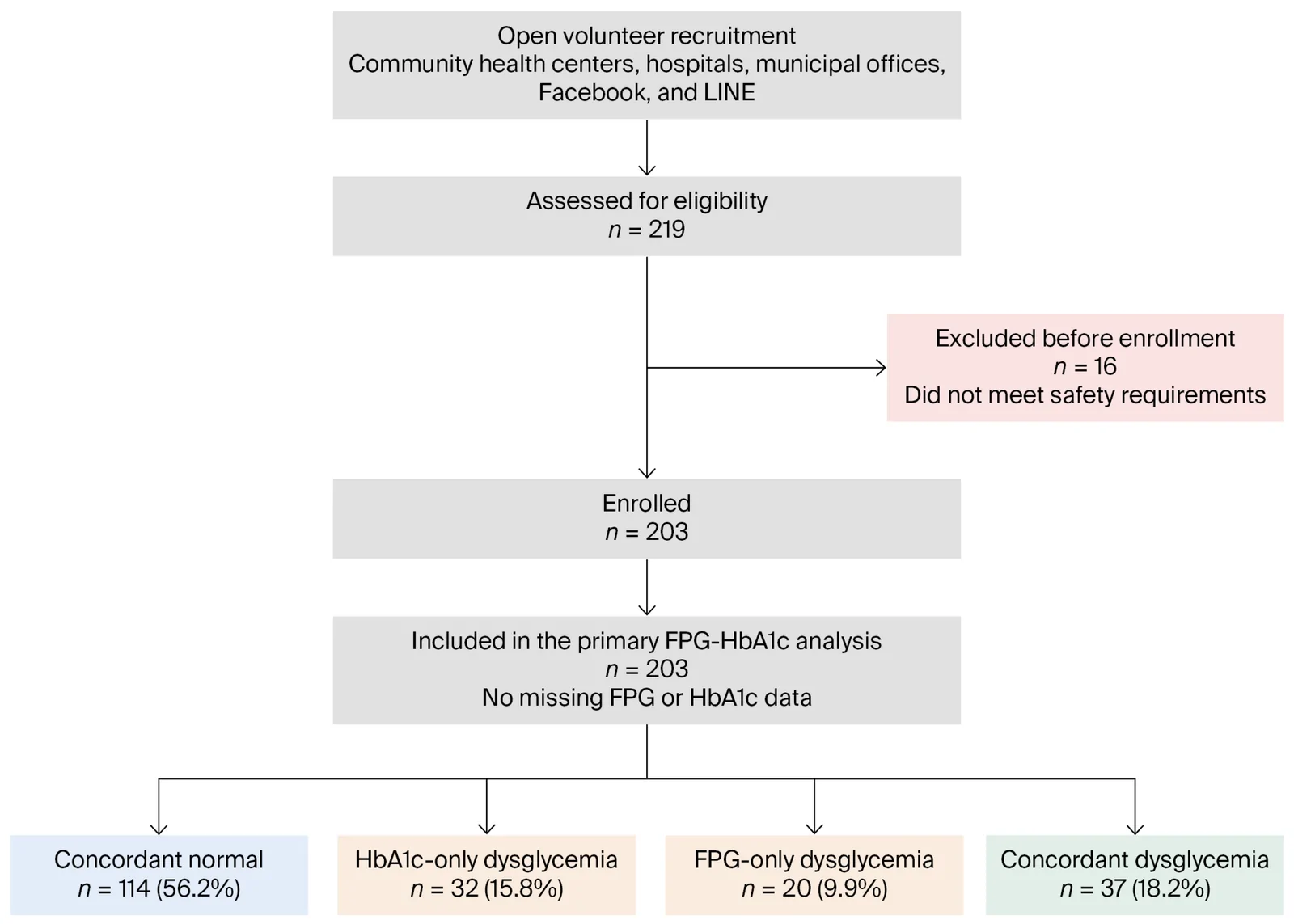
Flow diagram of participant recruitment, eligibility screening, enrollment, and classification according to concordance between fasting plasma glucose (FPG)- and glycated hemoglobin (HbA1c)-based glycemic categories. Participants with prediabetes-range or diabetes-range values were combined into the dysglycemia category for the binary phenotype classification. No enrolled participant had missing FPG or HbA1c data. Outcome-specific missing data are reported in the corresponding analyses.

**Figure 3:**
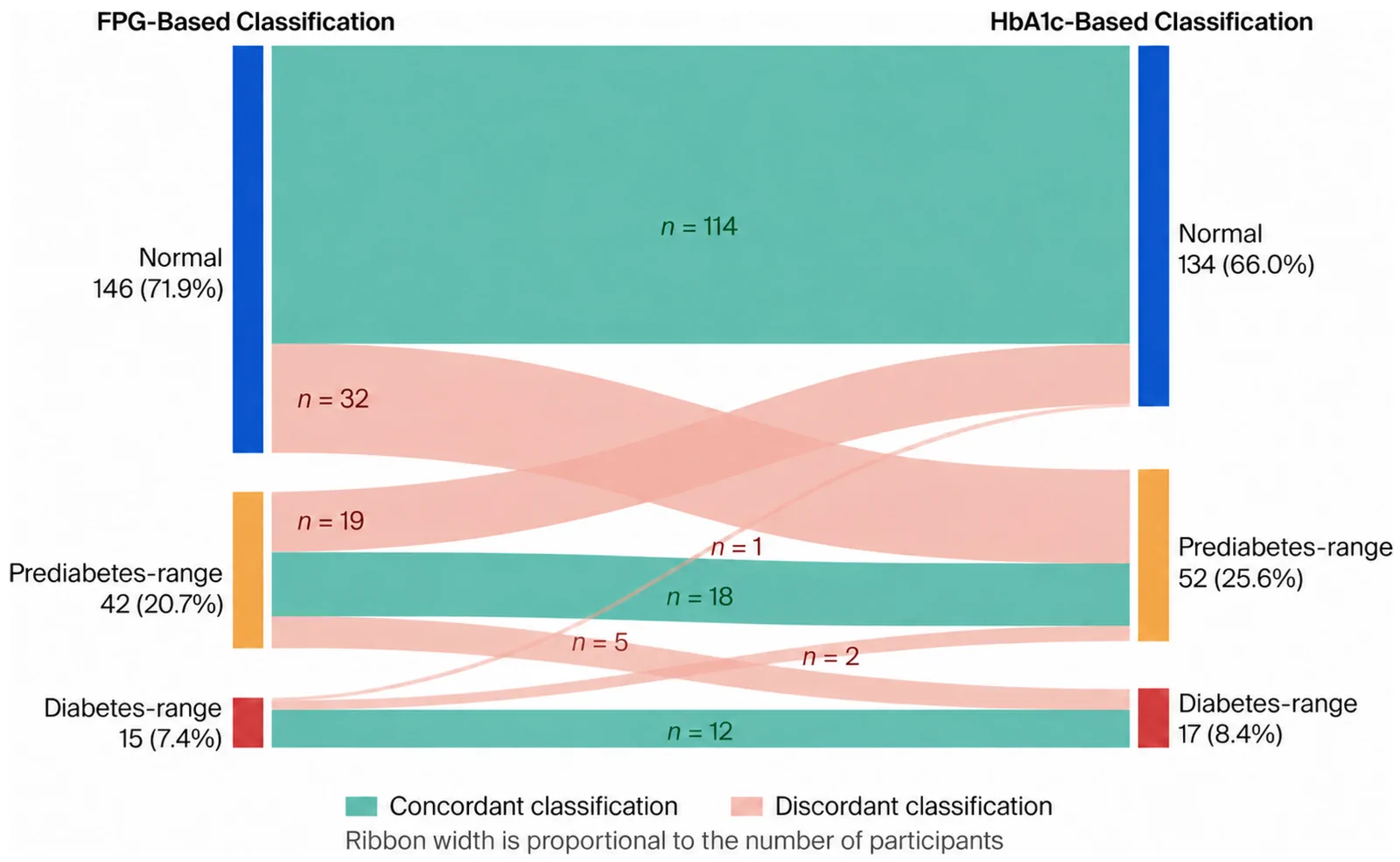
Alluvial diagram illustrating concordance and discordance between fasting plasma glucose (FPG)- and glycated hemoglobin (HbA1c)-based three-category glycemic classifications among 203 community-dwelling Thai older adults. Ribbon widths are proportional to the number of participants in each classification pathway. Green ribbons represent concordant classifications, whereas orange ribbons represent discordant classifications. Overall, 144 participants (70.9%) had concordant classifications, while 59 participants (29.1%) had discordant classifications.

**Figure 4:**
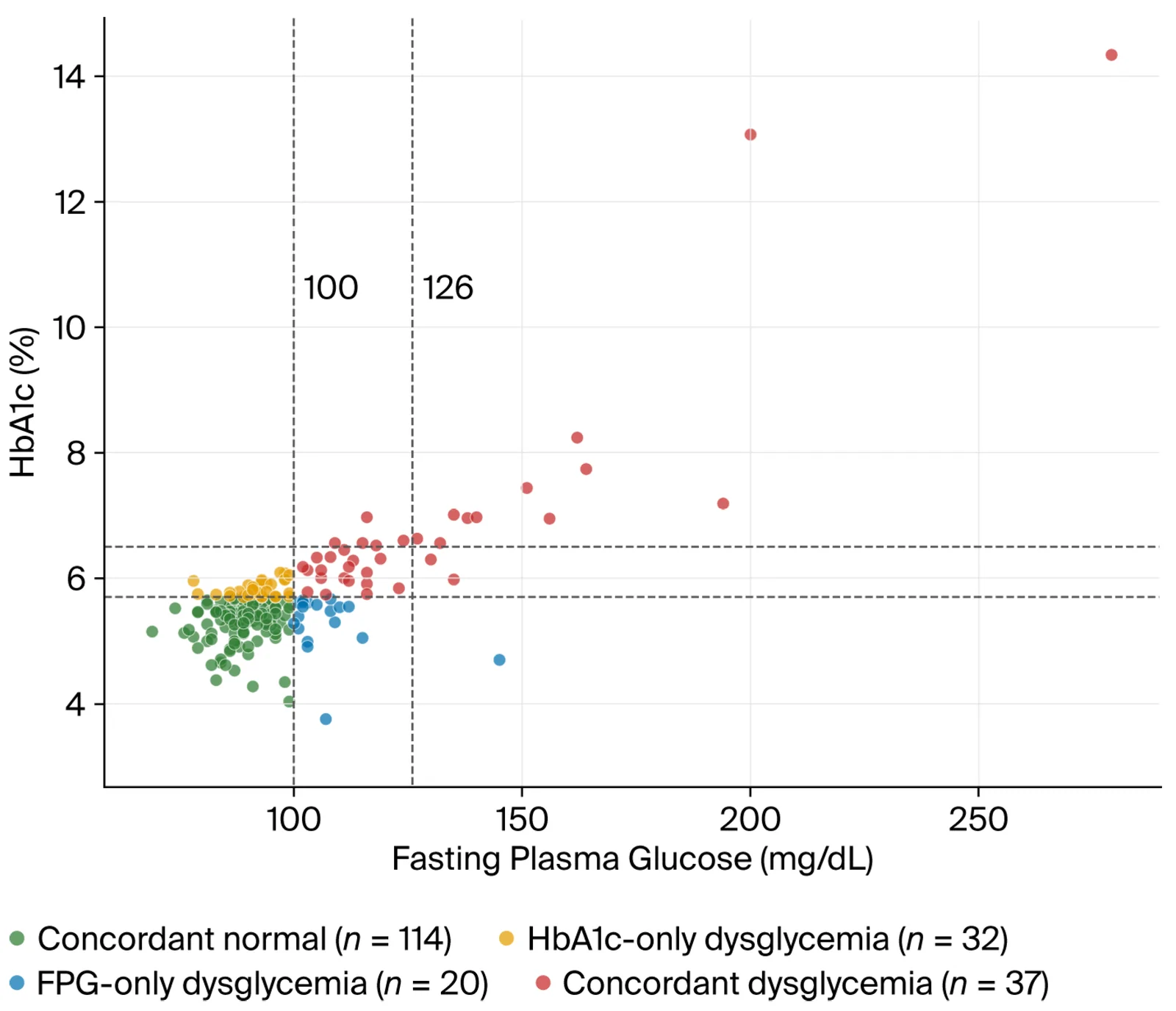
Scatterplot of fasting plasma glucose (FPG) and glycated hemoglobin (HbA1c) with classification thresholds at FPG 100 and 126 mg/dL and HbA1c 5.7% and 6.5%. Points are colored according to the four laboratory-defined binary phenotypes. The vertical dashed lines indicate FPG thresholds of 100 and 126 mg/dL; the horizontal dashed lines indicate HbA1c thresholds of 5.7% and 6.5%.

**Table 1:** Participant characteristics according to concordance between fasting plasma glucose- and HbA1c-based glycemic classifications.

Variables	Overall (*n* = 203)	Concordant Normal (*n* = 114)	HbA1c-Only Dysglycemia (*n* = 32)	FPG-Only Dysglycemia (*n* = 20)	Concordant Dysglycemia (*n* = 37)
Age (years)	66.00 (63.00–70.00)	66.00 (63.00–70.00)	65.00 (63.75–69.25)	67.50 (63.75–74.25)	66.00 (64.00–70.00)
Female, *n* (%)	167 (82.3)	92 (80.7)	28 (87.5)	14 (70.0)	33 (89.2)
BMI (kg/m^2^)	23.20 (21.35–25.85)	22.65 (20.82–24.88)	22.60 (21.30–24.68)	24.50 (22.73–27.95)	25.80 (23.70–28.20)
Waist circumference (cm)	82.00 (76.00–89.00)	80.00 (74.00–85.45)	80.50 (76.00–85.25)	90.00 (82.00–94.75)	90.00 (86.00–93.00)
Waist-to-hip ratio	0.86 (0.81–0.90)	0.84 (0.80–0.88)	0.84 (0.81–0.90)	0.89 (0.85–0.96)	0.90 (0.85–0.92)
SBP (mmHg)	127.00 (113.50–138.50)	123.50 (110.25–137.50)	128.00 (114.50–137.25)	128.00 (114.75–140.50)	131.00 (122.00–140.00)
DBP (mmHg)	74.00 (64.50–81.50)	72.50 (64.00–81.00)	77.50 (62.75–83.00)	78.00 (71.50–84.50)	74.00 (64.00–80.00)
HR (bpm)	70.0 (63.0–77.0)	69.0 (62.0–76.0)	69.0 (62.75–74.0)	66.5 (63.0–75.25)	71.0 (64.0–78.25)
PP (mmHg)	51.0 (43.0–61.0)	48.5 (42.0–60.0)	53.0 (44.0–59.0)	52.5 (42.75–57.25)	59.0 (47.0–66.0)
MAP (mmHg)	91.67 (81.83–99.67)	89.33 (80.67–98.58)	95.17 (80.25–100.67)	95.50 (87.00–101.17)	94.67 (82.00–101.00)
FPG (mg/dL)	93.00 (87.50–102.00)	89.00 (86.00–93.00)	93.00 (90.00–96.00)	103.00 (102.00–108.25)	116.00 (111.00–135.00)
HbA1c (%)	5.54 (5.29–5.79)	5.37 (5.15–5.49)	5.80 (5.74–5.93)	5.51 (5.16–5.61)	6.34 (6.09–6.96)
Insulin (µIU/mL)	7.93 (6.30–10.80)	7.01 (5.29–8.98)	8.99 (6.54–11.07)	8.68 (6.90–9.74)	11.20 (8.46–15.00)
Total cholesterol (mg/dL)	220.00 (196.00–249.00)	221.50 (206.25–252.25)	209.50 (184.00–240.25)	240.50 (214.75–279.50)	207.00 (176.00–223.00)
Triglycerides (mg/dL)	114.50 (87.50–150.75)	105.00 (81.00–139.50)	137.00 (110.00–166.50)	113.00 (95.00–159.50)	122.00 (100.00–182.00)
LDL-C (mg/dL)	130.00 (108.00–152.50)	135.00 (108.25–154.75)	113.00 (93.25–146.25)	139.00 (122.25–176.25)	123.00 (96.00–133.00)
HDL-C (mg/dL)	62.00 (54.00–72.00)	66.00 (57.00–76.00)	60.50 (53.50–66.75)	65.00 (55.25–72.00)	56.00 (51.00–66.00)
Known diabetes, *n* (%)	22 (10.8)	0 (0.0)	2 (6.2)	2 (10.0)	18 (48.6)
Glucose-lowering medication, *n* (%)	14 (6.9)	0 (0.0)	2 (6.2)	0 (0.0)	12 (32.4)
Antihypertensive medication, *n* (%)	44 (21.7)	14 (12.3)	12 (37.5)	4 (20.0)	14 (37.8)
Lipid-lowering medication, *n* (%)	52 (25.6)	18 (15.8)	14 (43.8)	6 (30.0)	14 (37.8)

Data are median (interquartile range) unless otherwise indicated. FPG- and HbA1c-based categories are laboratory test-result ranges from single measurements and are not confirmed diagnoses. Current diagnoses and medication use were self-reported. HR was available for 201 participants; other outcome-specific missingness is reflected in the corresponding analyses. BMI, body mass index; DBP, diastolic blood pressure; FPG, fasting plasma glucose; HbA1c, glycated hemoglobin; HDL-C, high-density lipoprotein cholesterol; HR, heart rate; LDL-C, low-density lipoprotein cholesterol; MAP, mean arterial pressure; PP, pulse pressure; SBP, systolic blood pressure.

**Table 2:** Agreement between fasting plasma glucose- and HbA1c-based glycemic classifications: three-category glycemic classification.

FPG-Based Classification	HbA1c Normal	HbA1c Prediabetes-Range	HbA1c Diabetes-Range	Total
Normal	**114**	32	0	146
Prediabetes-range	19	**18**	5	42
Diabetes-range	1	2	**12**	15
Total	134	52	17	203
Agreement statistic	Estimate	95% CI	*p*-value	
Exact agreement, %	70.9	–	–	
Unweighted Cohen’s κ	0.376	0.247–0.498	–	
Linear-weighted Cohen’s κ	0.486	0.355–0.601	–	
Bowker’s χ^2^ test	5.599	–	0.133	

Data in the cross-classification matrices are presented as numbers of participants. Bold values indicate concordant classifications. Normal, prediabetes-range, and diabetes-range classifications were defined as FPG < 100, 100–125, and ≥126 mg/dL, respectively, and HbA1c < 5.7%, 5.7–6.4%, and ≥6.5%, respectively. Cohen’s κ was used to quantify agreement beyond chance. Linear-weighted κ accounted for the ordinal distance between the three categories. Bowker’s test evaluated symmetry of three-category disagreement. CI, confidence interval; FPG, fasting plasma glucose; HbA1c, glycated hemoglobin.

**Table 3:** Agreement between fasting plasma glucose- and HbA1c-based glycemic classifications: binary glycemic classification.

FPG-Based Classification	HbA1c Normal	HbA1c Dysglycemia		Total
Normal	**114**	32		146
Dysglycemia	20	**37**		57
Total	134	69		203
Agreement statistic	Estimate	95% CI	*p*-value	
Exact agreement, %	74.4	–	–	
Cohen’s κ	0.404	0.268–0.533	–	
Continuity-corrected McNemar χ^2^	2.327	–	0.127	
Discordant classification, *n* (%)	52 (25.6%)	–	–	

Data in the cross-classification matrices are presented as numbers of participants. Bold values indicate concordant classifications. Dysglycemia was defined as FPG ≥ 100 mg/dL or HbA1c ≥ 5.7%, according to the corresponding classification method. Cohen’s κ was used to quantify agreement beyond chance. The continuity-corrected McNemar test evaluated directional disagreement in the binary classification. CI, confidence interval; FPG, fasting plasma glucose; HbA1c, glycated hemoglobin.

**Table 4:** Age- and sex-adjusted metabolic and hemodynamic profiles according to the FPG-HbA1c concordance phenotype.

Outcome	Overall *p*-Value	FDR *q*-Value	HbA1c-Only, B (95% CI)	*p*-Value; *q*-Value	FPG-Only, B (95% CI)	*p*-Value; *q*-Value	Concordant Dysglycemia, B (95% CI)	*p*-Value; *q*-Value
BMI (kg/m^2^)	<0.001	<0.001	0.26 (−1.02 to 1.53)	0.692; 0.765	2.52 (0.55 to 4.50)	0.012; 0.044	3.58 (2.15 to 5.01)	<0.001; <0.001
Waist circumference (cm)	<0.001	<0.001	1.33 (−1.84 to 4.49)	0.411; 0.538	7.95 (3.50 to 12.40)	<0.001; 0.003	9.88 (6.68 to 13.07)	<0.001; <0.001
Waist-to-hip ratio	<0.001	<0.001	0.01 (−0.01 to 0.04)	0.245; 0.340	0.05 (0.01 to 0.08)	0.011; 0.039	0.05 (0.03 to 0.07)	<0.001; <0.001
SBP (mmHg)	0.301	0.337	2.10 (−4.64 to 8.84)	0.541; 0.627	4.00 (−4.81 to 12.82)	0.373; 0.519	8.00 (−0.87 to 16.86)	0.077; 0.165
DBP (mmHg)	0.311	0.337	1.69 (−2.79 to 6.18)	0.459; 0.578	4.59 (−0.57 to 9.76)	0.081; 0.165	2.22 (−3.17 to 7.62)	0.419; 0.548
PP (mmHg)	0.252	0.337	0.41 (−3.69 to 4.50)	0.845; 0.881	−0.59 (−7.42 to 6.25)	0.866; 0.881	5.77 (−0.03 to 11.57)	0.051; 0.146
MAP (mmHg)	0.291	0.337	1.83 (−3.15 to 6.81)	0.472; 0.578	4.40 (−1.37 to 10.16)	0.135; 0.225	4.15 (−2.02 to 10.32)	0.188; 0.303
HR (bpm)	0.478	0.478	−1.08 (−4.48 to 2.32)	0.533; 0.612	0.15 (−4.92 to 5.22)	0.954; 0.962	2.36 (−1.47 to 6.19)	0.227; 0.340
Insulin (µIU/mL)	<0.001	0.002	1.52 (−0.04 to 3.08)	0.056; 0.151	2.71 (−0.63 to 6.05)	0.112; 0.194	6.16 (2.70 to 9.61)	<0.001; 0.003
Total cholesterol (mg/dL)	<0.001	<0.001	−14.38 (−31.90 to 3.14)	0.108; 0.189	26.91 (4.49 to 49.34)	0.019; 0.062	−26.46 (−42.31 to −10.60)	0.001; 0.005
Triglycerides (mg/dL)	0.004	0.007	24.69 (6.63 to 42.74)	0.007; 0.036	28.71 (−2.01 to 59.42)	0.067; 0.159	30.43 (7.98 to 52.87)	0.008; 0.036
LDL-C (mg/dL)	0.008	0.014	−14.41 (−30.91 to 2.10)	0.087; 0.165	18.90 (−2.21 to 40.01)	0.079; 0.165	−16.44 (−31.03 to −1.85)	0.027; 0.079
HDL-C (mg/dL)	0.003	0.007	−3.94 (−10.00 to 2.12)	0.203; 0.303	1.73 (−6.95 to 10.41)	0.696; 0.767	−9.01 (−14.03 to −3.98)	<0.001; 0.003

Regression coefficients (B) and 95% confidence intervals were estimated with age- and sex-adjusted linear regression using HC3 heteroscedasticity-consistent standard errors and concordant normal as the reference. The Benjamini–Hochberg procedure was applied separately across the 13 omnibus phenotype tests (overall FDR *q*-value column) and across the 39 phenotype-specific contrasts (*q*-values paired with contrast *p*-values). The two multiplicity families were interpreted separately; *q* < 0.05 indicated FDR significance. BMI, body mass index; CI, confidence interval; DBP, diastolic blood pressure; FDR, false discovery rate; HDL-C, high-density lipoprotein cholesterol; HR, heart rate; LDL-C, low-density lipoprotein cholesterol; MAP, mean arterial pressure; PP, pulse pressure; SBP, systolic blood pressure.

## Data Availability

The data are available upon request from the corresponding author. Restrictions apply to the availability of these data due to privacy and ethical considerations.

## Abbreviations

The following abbreviations are used in this manuscript:

BMI	Body mass index
BP	Blood pressure
CI	Confidence interval
CLIA	Chemiluminescence immunoassay
DBP	Diastolic blood pressure
EDTA	Ethylenediaminetetraacetic acid
FPG	Fasting plasma glucose
FDR	False-discovery-rate
HbA1c	Glycated hemoglobin
HDL-C	High-density lipoprotein cholesterol
HPLC	High-performance liquid chromatography
HR	Heart rate
IQR	Interquartile range
LDL-C	Low-density lipoprotein cholesterol
MAP	Mean arterial pressure
OGTT	Oral glucose tolerance test
PP	Pulse pressure
SBP	Systolic blood pressure
